# Complete Genome Sequence of Thermoactinomyces vulgaris Strain CDF, a Thermophilic Bacterium Capable of Degrading Chicken Feathers

**DOI:** 10.1128/MRA.00530-19

**Published:** 2019-07-11

**Authors:** Binbin Li, Feng Liu, Yuxia Ren, Yidi Ding, Yu Li, Xiao-Feng Tang, Bing Tang

**Affiliations:** a State Key Laboratory of Virology, College of Life Sciences, Wuhan University, Wuhan, China; University of Delaware

## Abstract

Thermoactinomyces vulgaris strain CDF was isolated from soil and shown to have the ability to degrade chicken feathers at high temperatures. Here, we report the complete genome sequence of this bacterium, which is 2,595,509 bp long with 2,642 predicted genes and an average G+C content of 48.14%.

## ANNOUNCEMENT

The members of the Thermoactinomyces genus are filamentous and thermophilic bacteria closely related to the genus Bacillus, and they produce endospores at the tips of the hyphae (1). The type species of the *Thermoactinomyces* genus is Thermoactinomyces vulgaris, which was initially isolated from decaying straw and manure (2). *Thermoactinomyces* species can spread their spores and hyphae into the air and may cause respiratory disorders such as lung hypersensitivity pneumonitis in humans and livestock (3). Due to their thermophilic nature, *Thermoactinomyces* species are generally used as a source of thermostable enzymes (4, 5). From the soil on the campus of Wuhan University in China, the extracellular protease-producing bacterium Thermoactinomyces vulgaris strain CDF (previously named *Thermoactinomyces* sp. CDF) was isolated on solid Luria-Bertani (LB) medium containing 1% skim milk at 55°C (6). The colonies were picked and restreaked until the strain was axenic, and the pure culture was deposited in the China Center for Type Culture Collection (CCTCC) under the accession number AB206328. Strain CDF has the ability to degrade chicken feathers, and three proteases of this bacterium have been previously characterized, including a spore-associated protease (6), a subtilisin-like keratinolytic protease (7), and a glutamyl endopeptidase (8).

Here we report the complete genome sequence of strain CDF. Genomic DNA was prepared by enzymatic lysis and phenol-chloroform extraction using an EasyPure bacterial DNA kit (TransGen Biotech, Beijing, China) and was used to construct a DNA library with SMRTbell template prep kit 1.0 (Pacific Biosciences). The library was constructed using BluePippin size selection (average fragment length, 13 kb; range, 5 to 36 kb) and was sequenced by single-molecule real-time (SMRT) sequencing on a PacBio RS II platform (9), generating 1,816 Mb of data from 117,832 filtered subreads with a mean length of 15.4 kb. The genome assembly was performed using the Hierarchical Genome Assembly Process version 3 (HGAP3) with the default settings (10). The final assembly yielded one chromosomal contig with a total length of 2,595,509 bp, a G+C content of 48.14%, and an average depth of coverage of 397×. Average nucleotide identity (ANI) analysis with Microbial Species Identifier (MiSI) (11) revealed a 99.53% match to the available reference draft genome of Thermoactinomyces vulgaris Gus 2-1 (GenBank accession number JPZM00000000) (12).

The genome was annotated by the NCBI Prokaryotic Genome Annotation Pipeline (PGAP) version 4.2 with the default settings (13). A total of 2,642 predicted genes were identified, including 2,498 protein-coding genes, 21 rRNA subunit genes (7 genes each for 5S, 16S, and 23S rRNA subunits), and 72 tRNA genes. The complete genome sequence of *T. vulgaris* CDF will be helpful in giving us a better understanding of the adaptation mechanism of thermophiles and to explore thermostable enzymes.

### Data availability.

The complete genome sequence of *Thermoactinomyces vulgaris* CDF has been deposited at DDBJ/ENA/GenBank under the accession number CP036487. Raw sequencing data have been deposited in the Sequence Read Archive (SRA) under the accession number SRX5581015.
